# Classification of Fashion Models’ Walking Styles Using Publicly Available Data, Pose Detection Technology, and Multivariate Analysis: From Past to Current Trendy Walking Styles

**DOI:** 10.3390/s24123865

**Published:** 2024-06-14

**Authors:** Yoshiyuki Kobayashi, Sakiko Saito, Tatsuya Murahori

**Affiliations:** 1 Human Augmentation Research Center, National Institute of Advanced Industrial Science and Technology (AIST), c/o Kashiwa II Campus, University of Tokyo, 6-2-3 Kashiwanoha, Kashiwa 277-0882, Japan; 2 Liberal Arts and Sciences, Nippon Institute of Technology, 4-1 Gakuendai, Saitama 345-8501, Japan; 3 TOKYO GAISHO Inc., #101 Heimat Daikanyama, 2-21-10 Ebisunishi, Tokyo 150-0021, Japan

**Keywords:** fashion model, gait, publicly available data, pose detection technology, multivariate analysis

## Abstract

Understanding past and current trends is crucial in the fashion industry to forecast future market demands. This study quantifies and reports the characteristics of the trendy walking styles of fashion models during real-world runway performances using three cutting-edge technologies: (a) publicly available video resources, (b) human pose detection technology, and (c) multivariate human-movement analysis techniques. The skeletal coordinates of the whole body during one gait cycle, extracted from publicly available video resources of 69 fashion models, underwent principal component analysis to reduce the dimensionality of the data. Then, hierarchical cluster analysis was used to classify the data. The results revealed that (1) the gaits of the fashion models analyzed in this study could be classified into five clusters, (2) there were significant differences in the median years in which the shows were held between the clusters, and (3) reconstructed stick-figure animations representing the walking styles of each cluster indicate that an exaggerated leg-crossing gait has become less common over recent years. Accordingly, we concluded that the level of leg crossing while walking is one of the major changes in trendy walking styles, from the past to the present, directed by the world’s leading brands.

## 1. Introduction

In the fashion industry, trend analysis is crucial to forecasting future market demands because trends change with the times [1]. Despite the importance given to the walking skills exhibited by models during fashion shows [2], trend analysis has not been applied to their walking styles. A Google Scholar search using the keywords “fashion model” and “gait analysis” returned only seven articles as of 30 May 2024. None of these studies focused on the walking styles of fashion models in a historical or evolutionary context. Fashion models need a high degree of technical athletic coordination to perfect their gaits on the runway [2]. Additionally, they need to understand sophisticated movement features that evolve based on the demands of the industry and society [2]. Hence, quantifying and reporting the features of trendy walking styles from the past to the present is extremely beneficial not only for models, their trainers, and agencies to increase models’ chances of being cast in runway shows, but also for designers and brands to sustain the development of the industry.

Until recently, it was virtually impossible to analyze the top performances of the world’s best performers, such as professional athletes, dancers, and fashion models, to understand the nature of their sophisticated techniques. However, such an analysis is now possible using three cutting-edge technologies: (1) video-sharing services, such as YouTube, in which high-resolution video resources (even at the 4K level) of the world’s best performers, including the recordings of the fashion shows of the world’s leading brands over the past 20 years, are available; (2) human pose detection technology, such as OpenPose [3], which is a novel sensing technology that can extract the skeletal coordinates of the human body from RGB images with remarkable accuracy [4]; (3) multivariate human-movement analysis techniques, which can classify the data into several groups based on the characteristics of whole-body movements [5,6]. Several researchers have used these technologies to evaluate the performances of the world’s best performers during actual competitions [7,8,9]. For example, Hobara et al. analyzed publicly available internet broadcasts to determine the running characteristics of able-bodied and amputee sprinters in actual 100 m races at world championships [7]. Recently, advanced pose detection technologies have been used in various industries, such as sports [10], healthcare [11], and entertainment [12]. Therefore, we concluded that the combination of these cutting-edge technologies, namely, the analysis of publicly available fashion show video resources using pose detection technology and multivariate analysis techniques, can clarify the characteristics of the sophisticated walking styles of the world’s leading fashion models that are modified over time based on the demands of the industry.

This study is the first to analyze the gaits of fashion models during actual fashion shows of the world’s leading brands using the abovementioned three cutting-edge technologies, aimed at quantifying and reporting the features of past and current trendy walking styles directed by the world’s leading brands. This study focused only on women’s fashion shows, as the market size of women’s apparel is 1.5 times larger than that of men’s apparel (USD 901.10 billion [13] vs. USD 568.90 billion [14] in 2023), and 77.7% of fashion models in the United States are women [15]. Furthermore, we focused only on (1) the spring/summer prêt-à-porter collections and (2) the first model to walk down the runway of each show. We took this approach because heavy clothing, often worn in autumn/winter, and haute-couture collections make skeletal detection less accurate with the current computer vision technology. Additionally, the media highlights the “first look” as the hottest model of the year, significantly reflecting the fashion trends of each period.

## 2. Materials and Methods

**Figure 1:**
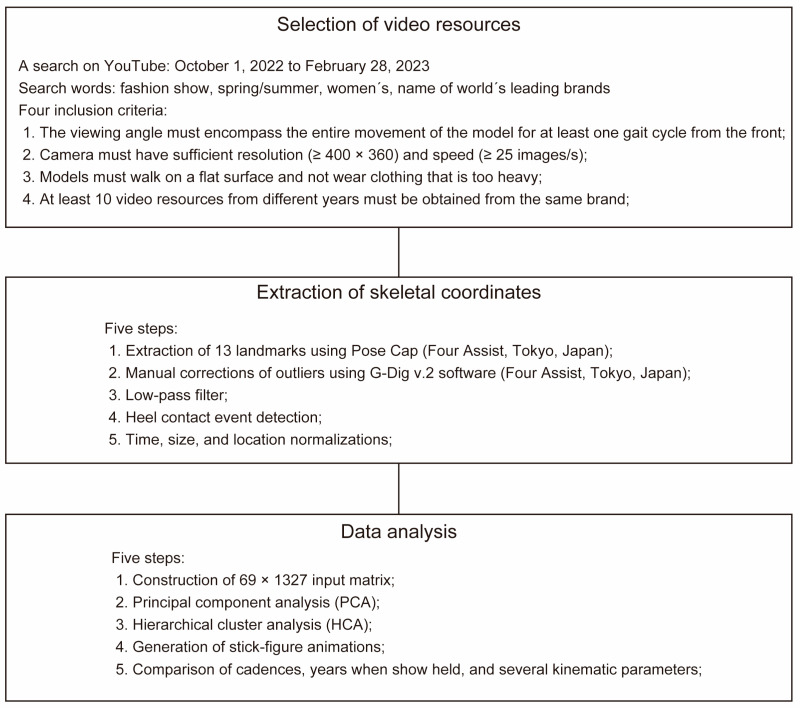
Flowchart of the overall research methodology.

A flowchart indicating the overall research methodology is shown in Figure 1.

### 2.1. Selection of Video Resources

The principal investigator of this research (Y.K.) performed a search on YouTube from 1 October 2022 to 28 February 2023. The search terms used were “fashion show”, “spring/summer”, and “women’s” combined with the names of brands (e.g., Versace, Dolce & Gabbana, and Louis Vuitton) participating in either one of the two most popular fashion weeks in the world (Paris or Milan) [16]. Manual selection of the videos was performed according to the following four inclusion criteria:

1. The viewing angle must encompass the entire movement of the model for at least one gait cycle from the front to avoid sliding effects;
2. The camera must have sufficient resolution (≥400 × 360) and speed (≥25 images/s);
3. Models must walk on a flat surface and not wear clothing that is too heavy, which would reduce the accuracy of the skeletal detection;
4. At least 10 video resources from different years must be obtained from the same brand so that transitions over time can be determined. This criterion was determined based on previous studies that conducted trend analyses in the fashion industry [17,18].

Because the walking environments among the shows could not be perfectly matched, two expert biomechanical researchers (Y.K. and S.S.) independently reviewed the videos and selected those that met the criteria. Finally, 69 videos were accepted, as listed in Table A1 (Appendix C).

### 2.2. Extraction of Skeletal Coordinates

After selecting the videos, the following five steps were performed to extract reliable skeletal coordinates of the models during a single gait cycle:

1. The 2D coordinates (x, y) of the following 13 landmarks were automatically extracted using the human pose detection library Pose Cap (Four Assist, Tokyo, Japan): head, shoulder center, hip center, right and left shoulders, right and left elbows, right and left hips, right and left knees, and right and left ankles. The default settings of the software were used for the pose detection. For skeletal landmarks with distinct outliers (e.g., when a part of the clothing was misidentified as a part of the human body), the principal investigator (Y.K.) made manual corrections using G-Dig v.2 software (Four Assist, Tokyo, Japan). The quality of the manual corrections was reviewed by a co-author (S.S.). We used this software because it allows for the manual correction of misidentified landmarks. This function was essential to the analysis of videos of fashion shows for which models sometimes wore flowy dresses;
2. A Butterworth low-pass filter with a cut-off frequency of 6 Hz (the default value in the biomechanical simulator OpenSim [19,20]) was used to smooth the time-series landmark signals;
3. For each video resource, Y.K. manually detected the timing of the right- and left-heel contact events frame by frame, and the skeletal coordinates for one gait cycle were extracted. When one gait cycle was extracted from the contact event of the left heel, the skeletal coordinates were inverted to the left and right for the subsequent analysis. Y.K. verified the accuracy of the heel contact event detection, and another biomechanics expert (S.S.) performed the same analysis on 18 randomly selected videos (25% of the total video resources). The mean absolute error of the manual heel contact event detection between the investigators was 0.53 frames (Table A2 in Appendix C);
4. Time, size, and location normalizations were performed for each data unit. For the time normalization, the skeletal coordinate data were linearly interpolated such that one gait cycle contained 51 frames (0–100%; 2% per frame). The cadence (steps/min), determined from the number of frames and the frame rate (images/sec) between the heel contact events, was also recorded for subsequent analyses. For the size normalization, the distance from the neck to the center of the hip was set to one, and the size of the entire body was adjusted. For the location normalization, the 2D coordinates of the hip joint at the first and last frames were both set to origin, and the skeletal coordinate data between events were linearly interpolated. These normalization processes were necessary because the time of one stride, the model size, and the walking locations varied among the models/video resources.

### 2.3. Data Analysis

The time-, size-, and location-normalized skeletal coordinate data, as well as the cadence data, were analyzed as follows:

1. A 69 × 1327 input matrix was constructed (69 models with 13 landmarks × 51 frames × 2D coordinates + cadence);
2. Principal component analysis (PCA) was applied to the input matrix using a correlation matrix to reduce the data dimension;
3. Hierarchical cluster analysis (HCA) was applied to the principal component scores (PCSs) of the principal component vectors (PCVs) with up to 80% cumulative variance to classify the data. The Euclidean distance and the Ward aggregation criterion were considered in the analysis. Dendrograms and cluster agglomeration schedules [21] were used to comprehensively determine the appropriate clusters for further analysis. Appendix A describes how to determine the appropriate clusters;
4. To help with the interpretation of the walking styles, stick-figure animations representing the walking style of each cluster were generated from the reconstructed skeletal coordinates. The skeletal coordinates were reconstructed from the mean PCS of each cluster in each PCV and the mean and standard deviations of each data unit, as performed in previous studies [5,22];
5. Furthermore, the cadences, years when the shows were held, and several kinematic parameters representing the walking styles of the clusters were compared statistically across the clusters. One-way analysis of variance (ANOVA) was applied when the normality and homoscedasticity assumptions were confirmed, and the Kruskal–Wallis test was applied when they were rejected. The Bonferroni method was used for multiple comparisons when a significant main effect was observed.

SPSS software (IBM SPSS Statistics v.19, IBM Inc., Armonk, NY, USA) was used for all statistical analyses. Given the number of data units in this study, we judged statistically significant differences by both *p*-values and effect sizes. This approach avoids the possible risk of misinterpreting the results based on the *p*-value alone. The effect sizes were the partial-eta-squared (*ŋ*^2^) value for the parametric tests and the r-value for the nonparametric tests. Based on previous studies [23,24], the criterion was set at *p* < 0.05 and a medium effect size (*ŋ*^2^ > 0.06 or *r* > 0.30).

## 3. Results

### 3.1. Classification of Walking Styles

**Figure 2:**
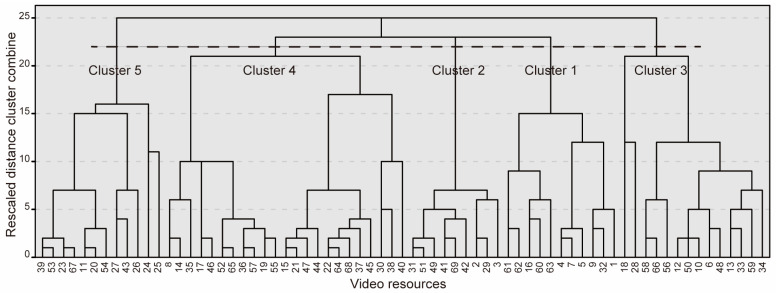
Dendrogram extracted using hierarchical cluster analysis. Based on the outputs and the detailed consideration described in Appendix A, we concluded that classifying the data into five clusters was the most appropriate for interpreting the results.

The PCA produced 57 PCVs with eigenvalues greater than one as outputs. Of the 57 PCVs, the first 13 PCVs explained more than 80% of the cumulative variance (Table A3 in Appendix C). Reliability of the PCA results have verified as described in Appendix B. The HCA produced the dendrogram shown in Figure 2 and the agglomeration schedule coefficients presented in Table A4 (Appendix C) as outputs. Based on these outputs and the detailed consideration described in Appendix A, we concluded that classifying the data into five clusters was the most appropriate for interpreting the results. Table 1 provides detailed information about each cluster.

### 3.2. Cadences and Years When Shows Were Held

For the cadence, normality was rejected. The Kruskal–Wallis test revealed no significant main effect on the cadence (K_(4, 69)_ = 2.892, not significant). For the years when the shows were conducted, both normality and homoscedasticity were rejected. The Kruskal–Wallis test revealed a significant main effect on the years when the shows were conducted (K_(4, 69)_ = 12.759, *p* = 0.013). Post hoc analyses revealed that the median year of a show was significantly earlier for Cluster 1 than it was for Cluster 5, with the medium effect size (*p* < 0.001, *r* = 0.41). The median (interquartile range (IQR)) cadence (steps/min) and the year when a show was conducted in each cluster are listed in Table 1.

### 3.3. Reconstructed Walking Styles

**Figure 3:**
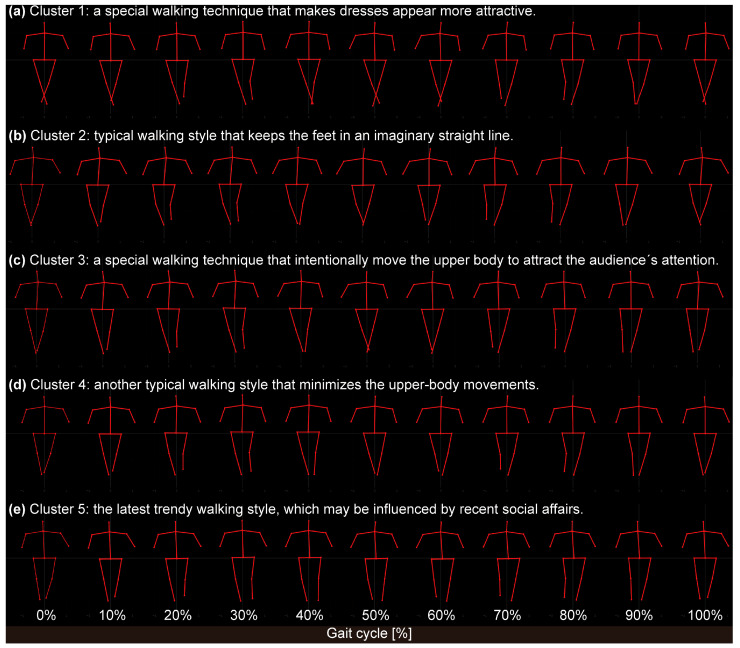
Reconstructed stick figures representing the walking styles of models in each cluster: (**a**) Cluster 1, (**b**) Cluster 2, (**c**) Cluster 3, (**d**) Cluster 4, and (**e**) Cluster 5. Each subfigure represents a consecutive 10% segment of the gait cycle. Full animation movies can be downloaded from Supplementary Material.

**Figure 4:**
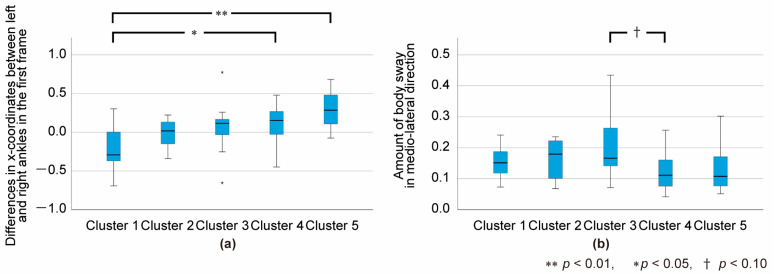
Box plot representing the gait features of each cluster: (**a**) medio-lateral distance between left- and right-ankle landmarks at the timing of heel contact; (**b**) amount of body sway in the medio-lateral direction. Pairs with significant or near-significant differences are marked in the figure.

Figure 3a–e show the skeletal coordinates representing the walking styles of each cluster. Each subfigure represents a consecutive 10% segment of the gait cycle. The full animation movies can be downloaded from the Supplementary Material. As shown, the stick figures in Cluster 1 crossed their legs most exaggeratedly, and the level of leg crossing decreased in the order of Clusters 2, 3, 4, and 5. In Clusters 4 and 5, the skeletal figures did not cross their legs. Therefore, we compared the medio-lateral distance between the left- and right-ankle landmarks in the first frame (at the timing of heel contact) among the clusters. After confirming both the normality and homoscedasticity of the data, a one-way ANOVA was applied. The results revealed a significant main effect with the large effect size (F_(4, 69)_ = 5.429, *p* < 0.001, *η*^2^ = 0.253), and multiple comparisons indicated significant differences between Clusters 1 and 4 (*p* < 0.05) and Clusters 1 and 5 (*p* < 0.01), as shown in Figure 4a.

The model in Cluster 3 tended to walk with a swaying upper body. Therefore, we compared the range of the neck landmark motion in the medio-lateral direction during the entire gait cycle among the clusters. Because the normality of the data was rejected, we applied the Kruskal–Wallis test. As a result, a significant main effect (K_(4, 69)_ = 9.794; *p* < 0.05) was confirmed. Multiple comparisons indicated near-significant differences between Clusters 3 and 4 with the medium effect size (*p* = 0.63, *r* = 0.33), as shown in Figure 4b.

## 4. Discussion

This study aimed to quantify and report the features of past and current trendy walking styles directed by the world’s leading brands. Therefore, we quantitatively analyzed the gaits of fashion models during real-world runway performances using publicly available video resources, human pose detection technology, and multivariate human-movement analysis techniques. Our results revealed that (1) the gaits of the fashion models analyzed in this study could be classified into five clusters; (2) the median year for the shows in each cluster became more recent in the order of Clusters 1, 2, 4, 3, and 5, with a significant difference observed between Clusters 1 and 5; and (3) the level of leg crossing has decreased in shows conducted more recently. Accordingly, we concluded that the level of leg crossing while walking is one of the major changes in trendy walking styles, from the past to the present, directed by the world’s leading brands. Detailed discussions of each cluster are described as follows.

### 4.1. Detailed Interpretations of Five Clusters

Cluster 1 was the oldest among the five clusters, comprising 11 videos from the early 2000s to the mid-2010s (Table 1). The reconstructed walking style clearly indicates that the models in Cluster 1 tended to walk with their legs crossed in the most exaggerated manner among the five clusters, with small trunk movements. According to the Fashion Republic forum [25], exaggerated cross-legged walking causes clothing to drape in a visually appealing, fluid manner. Indeed, several models classified in this cluster wore long skirts that moved along the models’ legs as they walked. Accordingly, the walking style of Cluster 1 can be interpreted as a special walking technique that makes dresses, such as long skirts, appear more attractive. However, these walking styles and fashions may not be recommended by the latest trends of the world’s leading brands, as evidenced by the fact that no shows after 2017 are included in this cluster.

Cluster 2 was the second oldest among the five clusters, and the smallest, with only nine videos, as listed in Table 1. The reconstructed stick figures indicate that the models in Cluster 2 tended to walk as if following a straight line. Guo et al. [26] stated that “keeping the feet in a straight line of imagination” is one of the basic requirements of walking for fashion models. Therefore, the walking style of Cluster 2 can be interpreted as one of the typical walking styles of fashion models who keep their feet in an imaginary straight line. Considering that the cluster is the smallest of the five and that no resources after 2020 are classified, the walking style of this cluster is likely becoming outdated.

Cluster 3 was the second most recent, with 14 videos included (Table 1). The reconstructed stick figures in this cluster are characterized by a large upper-body swing. Such a walking style is again another basic requirement of the walking style of fashion models, as described by Guo et al. [26]; that is, “upper-body relax, avoid swing range is too big”. However, in actual fashion shows, models seem to occasionally use their upper bodies to attract attention. Regarding the walking style of Karlie Kloss, one of the world’s most famous supermodels, the fashion magazine *Elle* [27] quoted, “Karlie uses her hips to sashay down the catwalk in heels with exaggerated arm movements to ensure all eyes are on her”. Therefore, the models included in this cluster may have intentionally moved their upper bodies to attract the audience’s attention. This type of walking is still applicable today as well because recent (2021, 2022, and 2023) shows are included in this cluster.

Cluster 4 was the third oldest and was the largest among the five clusters (Table 1). The reconstructed stick figures indicate that the models in this cluster tended to walk with the smallest upper-body motion among the five clusters. As mentioned in the previous paragraph, “upper-body relax, avoid swing range is too big” is described as one of the other basic requirements of the walking style of fashion models [26]. Therefore, the walking style of Cluster 4 can be interpreted as one of the other typical walking styles of fashion models that minimize upper-body movements. Because recent shows (2022 and 2023) are also included in this cluster, this type of walking is still applicable today as well.

Cluster 5 was the latest of the five clusters. A significant difference in the median years of the shows was observed between Cluster 1 and Cluster 5. This cluster had 12 videos, as listed in Table 1. Here, the models appeared to walk with a normal gait, not completely crossing their legs. From the mid-2010s, the keywords “gender neutral” or “gender fluidity” began to attract attention in the fashion scene [28]. Around the same period, “the charter for the well-being of fashion models” was released by an industry coalition [29]. As clothes and shoes become genderless, the gaits of models may also become genderless. Therefore, the walking style of Cluster 5 can be interpreted as the latest trend, which may be influenced by recent social affairs.

### 4.2. Limitations and Future Perspectives

Owing to the methodology employed, this study has several limitations. First, the image quality and camera angles were not consistent among the analyzed video resources. While considering the detailed criteria and validation methods employed to minimize the impacts of these factors, the reader should also keep these points in mind when interpreting the results. Furthermore, we only used software that utilizes fundamental two-dimensional human pose detection technology (Pose Cap and G-Dig v.2) because misidentified landmarks can be manually corrected. This function was essential for this study to analyze videos of fashion shows for which models sometimes wore flowy dresses. The use of quasi-three-dimensional (or 4D) pose detection technology, which has been recently developed [12,30,31], may provide further understanding of trendy walking styles. Recent studies on impressive walking styles have consistently reported that the pelvic postures in the sagittal plane also play an important role in the aesthetic impressions of gait [32,33,34].

The methodology employed also has some advantages. For example, the use of publicly available videos allow us to analyze the maximum performances of performers. They are conditioned to perform to their maximum potential at competitions and not in the laboratory. Additionally, it is difficult to mimic the atmosphere of a live environment (e.g., the excitement of an audience) in a laboratory setting. We will continue our research, applying the latest sensing technology, to provide beneficial information to fashion models who aspire to the top.

## Figures and Tables

**Table 1:** Detailed information for each cluster. For the cadence and years when the shows were held, the median (interquartile range) is reported. Statistical analyses revealed a significant main effect on the year when the show was held but not on the cadence.

	Cluster 1	Cluster 2	Cluster 3	Cluster 4	Cluster 5
Number of resources	11	9	14	23	12
Cadence	129.6 (9.6)	124.8 (16.8)	124.8 (9.6)	124.8 (4.8)	122.4 (13.2)
Years when shows were held	2006 (9) **	2011 (16)	2014.5 (15.5)	2014 (10)	2018.5 (5.5) **
Classified shows	D&G 2003	D&G 2005	D&G 2013	D&G 2015	D&G 2019
	D&G 2011	D&G 2009	D&G 2018	D&G 2022	LV 2009
	D&G 2012	VAL 1998	D&G 2020	D&G 2023	LV 2018
	D&G 2014	VAL 2001	D&G 2021	LV 2004	LV 2019
	D&G 2017	VAL 2018	LV 2006	LV 2008	LV 2020
	LV 2003	VAL 2020	VAL 1995	LV 2015	LV 2021
	VAL 2002	VER 2011	VAL 2004	LV 2016	LV 2022
	SL 2003	VER 2013	VAL 2005	VAL 1999	VAL 2015
	SL 2004	SL 2020	VER 2004	VAL 2010	VAL 2021
	SL 2006		VER 2012	VAL 2012	VER 2015
	SL 2008		VER 2019	VAL 2013	VER 2016
			VER 2022	VAL 2014	SL 2017
			VER 2023	VAL 2017	
			SL 2016	VAL 2022	
				VAL 2023	
				VER 2000	
				VER 2002	
				VER 2014	
				VER 2017	
				VER 2021	
				SL 2009	
				SL 2012	
				SL 2019	

D&G: Dolce & Gabbana; LV: Louis Vuitton; SL: Saint Laurent; VAL: Valentino; VER: Versace. ** Significant differences (*p* < 0.05) between the clusters with asterisks.

## Data Availability

The datasets generated and/or analyzed in the current study can be obtained from the corresponding author upon reasonable request.

## Supplementary Materials

The following supporting information can be downloaded at https://www.mdpi.com/article/10.3390/s24123865/s1, Video S1: CLST1_Animation.mp4, Video S2: CLST2_Animation.mp4, Video S3: CLST3_Animation.mp4, Video S4: CLST4_Animation.mp4, Video S5: CLST1_Animation.mp5.

## Appendix A. Detailed Consideration of Clusters

Considering insufficient clusters made from only one or two resources and understanding their characteristics was inconsequential to achieve the study objectives. We therefore set the minimum number of data units to be included in each cluster at 7, which is approximately 10% of the total number of data units analyzed (i.e., 69 in total). As previous studies have and several lectures have instructed, we determined the optimal number of clusters comprehensively from the percent changes of the agglomeration schedule coefficients at each stage, in combination with the visual inspection of the dendrogram [37,38,39].

A hierarchical cluster analysis produced the dendrogram shown in Figure 1 and the agglomeration schedule coefficients presented in Table A2 (Appendix C) as outputs. The greatest percent change was found between stages 62 and 63, which indicated that classification into six clusters was optimal. However, in this case, Cluster 6 consists of only two shows (Louis Vuitton 2006 and Valentino 1995), violating the criterion for the minimum number of data units to be included in each cluster. Therefore, we considered classifying the data into five clusters. In this case, all the clusters met the criterion, and the visual inspection of the dendrogram was reasonable as well. Consequently, we concluded that classifying the data into five clusters was considered the most appropriate for interpreting the results.

## Appendix B. Verification of the Reliability of the PCA Results

To verify the reliability of the PCA results in this study, we conducted the following test:

1. Select 50 data units randomly from 69 data units to create a data subset;
2. Repeat procedure 1 to create five different data subsets;
3. Apply PCA to each of the data subsets;
4. Calculate the correlation coefficients between the original and subset principal component loadings for principal components 1–13.

As a result, the average correlation coefficients for the five datasets were greater than 0.3 for principal components 1–13. This suggests that similar results could have been obtained from the PCA even if different datasets were used. Therefore, we can conclude that the results of the PCA reported in our study are reliable.

## Appendix C. Supplemental Tables

**Table A1:** List of selected resources. SS: spring/summer.

	Brand	Year/Season	Location	Link (All Links Accessed on 31 May 2024)
1	Dolce & Gabbana	2003 SS	Milan	https://www.youtube.com/watch?v=p_nZpTBYsaA
2	Dolce & Gabbana	2005 SS	Milan	https://www.youtube.com/watch?v=fsbW4GqRJN0
3	Dolce & Gabbana	2009 SS	Milan	https://www.youtube.com/watch?v=QTXiOStGz5w
4	Dolce & Gabbana	2011 SS	Milan	https://www.youtube.com/watch?v=g0_Y9CuETcM
5	Dolce & Gabbana	2012 SS	Milan	https://www.youtube.com/watch?v=kuJ4lIR99fs
6	Dolce & Gabbana	2013 SS	Milan	https://www.youtube.com/watch?v=iB2ZuXC7da0
7	Dolce & Gabbana	2014 SS	Milan	https://www.youtube.com/watch?v=l6PaWaREPPo
8	Dolce & Gabbana	2015 SS	Milan	https://www.youtube.com/watch?v=bTT3bWCPfxc
9	Dolce & Gabbana	2017 SS	Milan	https://www.youtube.com/watch?v=CPZmkiUsATU
10	Dolce & Gabbana	2018 SS	Milan	https://www.youtube.com/watch?v=n986zYUsDfg
11	Dolce & Gabbana	2019 SS	Milan	https://www.youtube.com/watch?v=T-xUKG_rUGA
12	Dolce & Gabbana	2020 SS	Milan	https://www.youtube.com/watch?v=I2AbpKwRR40
13	Dolce & Gabbana	2021 SS	Milan	https://www.youtube.com/watch?v=rVjAtPiW3MQ
14	Dolce & Gabbana	2022 SS	Milan	https://www.youtube.com/watch?v=RjIyaVwgryE
15	Dolce & Gabbana	2023 SS	Milan	https://www.youtube.com/watch?v=Gpvgfc1mmvo
16	Louis Vuitton	2003 SS	Paris	https://www.youtube.com/watch?v=LHDaKrpiV9o
17	Louis Vuitton	2004 SS	Paris	https://www.youtube.com/watch?v=KN_AaQqYHpo
18	Louis Vuitton	2006 SS	Paris	https://www.youtube.com/watch?v=a21WpJJDods
19	Louis Vuitton	2008 SS	Paris	https://www.youtube.com/watch?v=Bx8iBjohtig
20	Louis Vuitton	2009 SS	Paris	https://www.youtube.com/watch?v=JGPlKM-gDGI
21	Louis Vuitton	2015 SS	Paris	https://www.youtube.com/watch?v=c3ZDGxxgCao
22	Louis Vuitton	2016 SS	Paris	https://www.youtube.com/watch?v=IK_9fSslijU
23	Louis Vuitton	2018 SS	Paris	https://www.youtube.com/watch?v=k57CBLm8jCs
24	Louis Vuitton	2019 SS	Paris	https://www.youtube.com/watch?v=PJleMAHoKek
25	Louis Vuitton	2020 SS	Paris	https://www.youtube.com/watch?v=BKy5SJjKTZ0
26	Louis Vuitton	2021 SS	Paris	https://www.youtube.com/watch?v=6G7L4rpxQfI
27	Louis Vuitton	2022 SS	Paris	https://www.youtube.com/watch?v=D7NYdae-KlA
28	Valentino	1995 SS	Paris	https://www.youtube.com/watch?v=g0DArKBQrdY
29	Valentino	1998 SS	Paris	https://www.youtube.com/watch?v=cACLF2Jqr6A
30	Valentino	1999 SS	Paris	https://www.youtube.com/watch?v=A8SnyF7Wqpw
31	Valentino	2001 SS	Paris	https://www.youtube.com/watch?v=jffPfbM0Nok
32	Valentino	2002 SS	Paris	https://www.youtube.com/watch?v=OLCl_o7a3so
33	Valentino	2004 SS	Paris	https://www.youtube.com/watch?v=6gR-f0pxmjQ
34	Valentino	2005 SS	Paris	https://www.youtube.com/watch?v=yAboBLAJMmQ
35	Valentino	2010 SS	Paris	https://www.youtube.com/watch?v=9OAyyZqPcFg
36	Valentino	2012 SS	Paris	https://www.youtube.com/watch?v=QtpYH3qnB0k
37	Valentino	2013 SS	Paris	https://www.youtube.com/watch?v=WVuRiuI3wag
38	Valentino	2014 SS	Paris	https://www.youtube.com/watch?v=BqgX5C_k8Ps
39	Valentino	2015 SS	Paris	https://www.youtube.com/watch?v=4PenthS8Pmw
40	Valentino	2017 SS	Paris	https://www.youtube.com/watch?v=RidHtqKqz78
41	Valentino	2018 SS	Paris	https://www.youtube.com/watch?v=LR2qbYb_s2A
42	Valentino	2020 SS	Paris	https://www.youtube.com/watch?v=pSkfcbzebSc
43	Valentino	2021 SS	Milan	https://www.youtube.com/watch?v=RktAHdMZOAs
44	Valentino	2022 SS	Paris	https://www.youtube.com/watch?v=ZKofPZM2ipw
45	Valentino	2023 SS	Paris	https://www.youtube.com/watch?v=B4jrkcZP0vQ
46	Versace	2000 SS	Milan	https://www.youtube.com/watch?v=l-zCHv6gwnE
47	Versace	2002 SS	Milan	https://www.youtube.com/watch?v=c77CR_VYKsQ
48	Versace	2004 SS	Milan	https://www.youtube.com/watch?v=Mw4ieZtKjUY
49	Versace	2011 SS	Milan	https://www.youtube.com/watch?v=BypOv68hdBk
50	Versace	2012 SS	Milan	https://www.youtube.com/watch?v=waJjDVWGPPg
51	Versace	2013 SS	Milan	https://www.youtube.com/watch?v=tPLS2dR6Z10
52	Versace	2014 SS	Milan	https://www.youtube.com/watch?v=A3OJYERxFpI
53	Versace	2015 SS	Milan	https://www.youtube.com/watch?v=HmPTucTXyyY
54	Versace	2016 SS	Milan	https://www.youtube.com/watch?v=yeXZLSqUBqo
55	Versace	2017 SS	Milan	https://www.youtube.com/watch?v=KnDwRS-opLI
56	Versace	2019 SS	Milan	https://www.youtube.com/watch?v=SFdYNuWmnqo
57	Versace	2021 SS	Milan	https://www.youtube.com/watch?v=Ffm-DfOTtv8
58	Versace	2022 SS	Milan	https://www.youtube.com/watch?v=zsu2WRFaUoQ
59	Versace	2023 SS	Milan	https://www.youtube.com/watch?v=hoKDrFyQDy0
60	Saint Laurent	2003 SS	Paris	https://www.youtube.com/watch?v=eAtT8PGQuFw
61	Saint Laurent	2004 SS	Paris	https://www.youtube.com/watch?v=rha577L91Qs
62	Saint Laurent	2006 SS	Paris	https://www.youtube.com/watch?v=E1Lxk9Sylng
63	Saint Laurent	2008 SS	Paris	https://www.youtube.com/watch?v=AId0Za9azmw
64	Saint Laurent	2009 SS	Paris	https://www.youtube.com/watch?v=U2ebRPcvFk0
65	Saint Laurent	2012 SS	Paris	https://www.youtube.com/watch?v=QFbGAWyWQ_c
66	Saint Laurent	2016 SS	Paris	https://www.youtube.com/watch?v=JbQL-wkKuq0
67	Saint Laurent	2017 SS	Paris	https://www.youtube.com/watch?v=IdRQ4E9N2Oc
68	Saint Laurent	2019 SS	Paris	https://www.youtube.com/watch?v=gtSbb-Euswk
69	Saint Laurent	2020 SS	Paris	https://www.youtube.com/watch?v=RwxyYras96k

**Table A2:** Validation of manually detected heel contact events.

		HC1: Y.K.	HC2: Y.K.	HC1: S.S.	HC2: S.S.	HC1	HC2
ID	Resources	Frame Number	Frame Number	Frame Number	Frame Number	Absolute Difference	Absolute Difference
1	Dolce & Gabbana 2003	16	43	16	43	0	0
6	Dolce & Gabbana 2013	21	46	21	46	0	0
8	Dolce & Gabbana 2015	18	43	17	43	1	0
9	Dolce & Gabbana 2017	27	54	27	54	0	0
14	Dolce & Gabbana 2022	14	36	14	38	0	2
16	Louis Vuitton 2003	25	49	26	49	1	0
18	Louis Vuitton 2006	25	49	24	48	1	1
21	Louis Vuitton 2015	25	51	26	51	1	0
30	Valentino 1998	30	57	29	56	1	1
34	Valentino 2005	32	59	32	59	0	0
36	Valentino 2012	17	43	18	43	1	0
39	Valentino 2015	15	42	16	42	1	0
41	Valentino 2018	18	43	18	43	0	0
43	Valentino 2021	21	46	20	45	1	1
49	Versace 2011	12	36	12	36	0	0
51	Versace 2013	14	39	14	39	0	0
54	Versace 2016	16	40	17	41	1	1
68	Saint Laurent 2019	16	41	14	39	2	2

HC: heel contact.

**Table A3:** Total variance of principal components.

Principal Component	Eigenvalues			Principal Component	Eigenvalues		
	Total	% of Variance	Cumulative %		Total	% of Variance	Cumulative %
1	261.631	19.776	19.776	31	4.733	0.358	94.764
2	200.914	15.186	34.962	32	4.315	0.326	95.090
3	182.836	13.82	48.782	33	4.228	0.320	95.409
4	101.47	7.670	56.451	34	4.013	0.303	95.713
5	68.524	5.179	61.631	35	3.877	0.293	96.006
6	52.543	3.972	65.602	36	3.421	0.259	96.264
7	44.647	3.375	68.977	37	3.241	0.245	96.509
8	39.104	2.956	71.933	38	3.019	0.228	96.738
9	31.303	2.366	74.299	39	2.892	0.219	96.956
10	28.842	2.180	76.479	40	2.788	0.211	97.167
11	22.418	1.694	78.173	41	2.702	0.204	97.371
12	19.439	1.469	79.643	42	2.532	0.191	97.563
13	19.056	1.440	81.083	43	2.502	0.189	97.752
14	17.008	1.286	82.368	44	2.324	0.176	97.927
15	16.480	1.246	83.614	45	2.153	0.163	98.090
16	15.797	1.194	84.808	46	1.977	0.149	98.240
17	14.927	1.128	85.936	47	1.830	0.138	98.378
18	13.764	1.040	86.977	48	1.756	0.133	98.511
19	12.733	0.962	87.939	49	1.734	0.131	98.642
20	10.693	0.808	88.747	50	1.606	0.121	98.763
21	9.769	0.738	89.486	51	1.52	0.115	98.878
22	9.515	0.719	90.205	52	1.431	0.108	98.986
23	8.678	0.656	90.861	53	1.344	0.102	99.088
24	8.190	0.619	91.480	54	1.273	0.096	99.184
25	8.062	0.609	92.089	55	1.137	0.086	99.27
26	7.134	0.539	92.629	56	1.112	0.084	99.354
27	6.474	0.489	93.118	57	1.050	0.079	99.433
28	5.943	0.449	93.567				
29	5.658	0.428	93.995				
30	5.441	0.411	94.406				

**Table A4:** Agglomeration schedule provided by HCA.

Stage	Combined Clusters		Coefficients	Stage at Which the Cluster First Appears		Next Stage
	Cluster 1	Cluster 2		Cluster 1	Cluster 2	
1	39	53	0.994	0	0	16
2	22	64	2.608	0	0	11
3	31	51	4.603	0	0	15
4	23	67	6.677	0	0	16
5	52	65	9.029	0	0	32
6	15	21	11.692	0	0	19
7	11	20	14.360	0	0	22
8	36	57	17.112	0	0	29
9	19	55	20.687	0	0	29
10	12	50	24.324	0	0	17
11	22	68	28.195	2	0	28
12	41	69	32.124	0	0	35
13	17	46	36.383	0	0	53
14	4	7	40.649	0	0	23
15	31	49	44.916	3	0	37
16	23	39	49.184	4	1	48
17	10	12	53.572	0	10	38
18	58	66	58.131	0	0	42
19	15	47	62.808	6	0	24
20	8	14	67.498	0	0	44
21	2	29	72.260	0	0	41
22	11	54	77.202	7	0	48
23	4	5	82.210	14	0	58
24	15	44	87.270	19	0	46
25	61	62	92.967	0	0	50
26	9	32	98.941	0	0	36
27	13	33	105.013	0	0	39
28	22	37	111.342	11	0	31
29	19	36	117.783	9	8	32
30	6	48	124.437	0	0	38
31	22	45	131.447	28	0	46
32	19	52	138.884	29	5	53
33	16	60	146.627	0	0	43
34	27	43	154.417	0	0	49
35	41	42	162.358	12	0	37
36	1	9	171.666	0	26	58
37	31	41	181.129	15	35	47
38	6	10	191.068	30	17	51
39	13	59	201.125	27	0	45
40	30	38	211.187	0	0	52
41	2	3	221.706	21	0	47
42	56	58	232.444	0	18	57
43	16	63	243.376	33	0	50
44	8	35	254.721	20	0	54
45	13	34	267.346	39	0	51
46	15	22	280.375	24	31	62
47	2	31	293.487	41	37	65
48	11	23	306.696	22	16	59
49	26	27	320.456	0	34	59
50	16	61	337.771	43	25	60
51	6	13	355.717	38	45	57
52	30	40	374.133	40	0	62
53	17	19	392.680	13	32	54
54	8	17	412.592	44	53	63
55	24	25	433.653	0	0	61
56	18	28	455.566	0	0	64
57	6	56	478.205	51	42	64
58	1	4	501.157	36	23	60
59	11	26	530.219	48	49	61
60	1	16	559.464	58	50	65
61	11	24	590.135	59	55	67
62	15	30	621.827	46	52	63
63	8	15	660.884	54	62	66
64	6	18	700.994	57	56	68
65	1	2	744.246	60	47	66
66	1	8	788.376	65	63	67
67	1	11	835.548	66	61	68
68	1	6	884.000	67	64	0
